# Epilepsy presenting only with severe abdominal pain

**DOI:** 10.4103/1817-1745.76123

**Published:** 2010

**Authors:** Nikolina Zdraveska, Aco Kostovski

**Affiliations:** Department for Gastroenterohepatology, University Children’s Hospital, Skopje, Macedonia

Dear Sir,

Abdominal epilepsy (AE) is an uncommon syndrome in which gastrointestinal complaints, mostly abdominal pain, are a result of a seizure activity.

It is characterized by paroxysmally diverse abdominal symptoms, definite EEG abnormalities and favourable response to the introduction of epilepsy drugs[1] Gastrointestinal signs and symptoms may occur as the sole manifestation of a simple partial seizure or as the initial manifestation of a complex partial seizure. In the absence of impaired consciousness, the epileptic cause of these episodes can be difficult to diagnose and may lead to exhaustive gastrointestinal investigation. We report a patient with recurrent episodes of severe abdominal pain, without obvious associated symptoms suggestive of central nervous system (CNS) abnormalities, but with EEG abnormalities and a positive response to anticonvulsive therapy.

A 14-year-old girl was referred to our hospital because of attacks of recurrent abdominal pain for the past 4 years. She was born after uncomplicated pregnancy and delivery and she had normal development and schooling. There was no prior significant illness. The father of the child suffered from peptic ulcer.

The pain was colicky and paroxysmal in nature, distributed mainly in the epigastric region; it was nonradiating and had no apparent relationship with meals. This intense pain was almost always accompanied with pallor and dizziness. Several times, she reported occurrence of nausea, vomiting and diarrhea after these attacks of pain. There was no alteration of consciousness and she had not experienced headaches. She never had convulsions. Each episode used to last for 10–30 min, with spontaneous resolution of symptoms, and recurred three to four-times a month. During the examination at gastroenterology unit the girl was treated with analgesics, antihistaminic (H2 blockers) and unspecific and placebo therapy such as vitamin B6 without any clinical improvement.

Physical examination, including neurological status, was normal. Laboratory studies were within normal limits, including complete blood count, liver function tests, amylase, *Helicobacter pylori* IgG titer, stool examinations for ova and parasites. Abdominal ultrasound and upper gastrointestinal endoscopy were normal.

**Figure 1:**
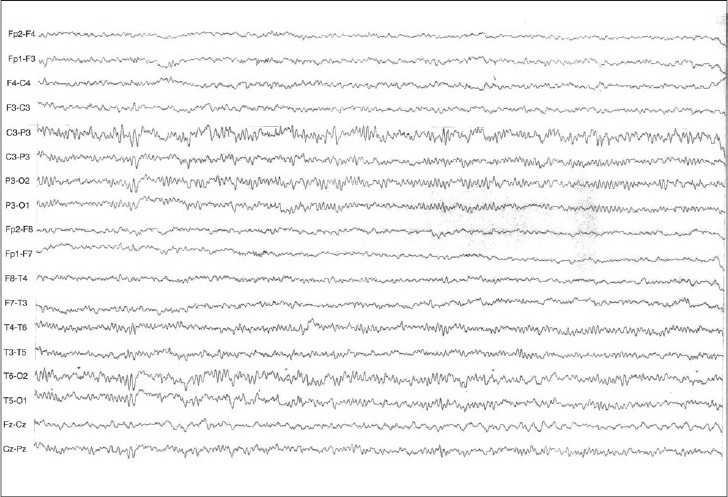
EEG record showing sharp waves and spikes from the right centrotemporal region

**Figure 2:**
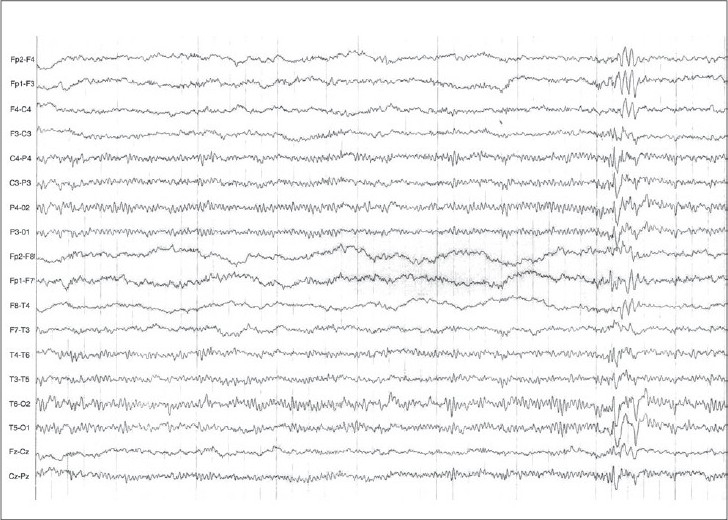
EEG recorded on a regular ambulatory check up after introducing carbamazepine treatment. Sharp waves and spikes are seen over the right temporal region with secondary generalization

She underwent an EEG examination, which revealed repetitive spikes, sharp waves over the right central and temporal electrodes with secondary generalization [Figures 1, 2]. Magnetic resonance imaging of the brain was performed (1-tesla field strength MRI- T1, T2 sequences plus FLAIR) which failed to detect any structural anomalies. The child was diagnosed as having temporal lobe – “abdominal” epilepsy – and treatment with carbamazepine was initiated. This was followed by a significant clinical improvement, and she has been asymptomatic during the following 2 years of follow-up.

Recurrent episodes of abdominal pain are common in childhood. In a minority of patients in which an abdominal pathology is excluded, a neurological cause should be considered. Among the diagnostic possibilities are migraine and AE.[2] Pain as an ictal symptom – distinct from other sensory phenomena – is a rare epileptic feature. In Young and Blume’s study out of 858 epileptic patients, only 24 (2.8%) experienced pain as a prominent part of their seizures. Most of them reported headaches (11 of 24), or unilateral face and body pain (10 of 24). Only 3 of them or 0.3% of their patients with epilepsy had ictal abdominal pain.[3] Abdominal epileptic pain was usually described as a severe and sharp sensation (“like a knife”), mostly in the periumbilical localization, but it was also experienced in the whole abdomen or in just one quadrant of the abdomen with a variable duration.[3 4]

Focal epilepsy presenting with gastrointestinal symptoms is now considered a definite clinical entity in the semiological seizure classification.[5] A review of the history of this syndrome yielded 36 cases reported in the English literature in the past 34 years.[1]

The pathophysiology of abdominal epilepsy remains unclear. Several mechanisms relating brain electrical activity to abdominal pain have been suggested. One of the possible explanations is that temporal lobe seizure activity usually arises in or involves the amygdala. Therefore the patients who have the temporal lobe epilepsy may have gastrointestinal symptoms, since discharges arising in the amygdala can be transmitted to the gut via dense direct projections to the dorsal motor nucleus of the vagus. In addition, sympathetic pathways from the amygdala to the gastrointestinal tract can be activated via the hypothalamus.

Criteria for the diagnosis of AE are: (1) otherwise unexplained, paroxysmal gastrointestinal complaints, (2) symptoms of a CNS disturbance; (3) an abnormal EEG with findings specific for a seizure disorder and (4) improvement with anticonvulsant drugs. Gastrointestinal manifestations include recurrent abdominal pain, nausea, vomiting, bloating and diarrhea, and a similar diversity of CNS manifestations has also been reported, including confusion, fatigue, headache, dizziness and syncope.

In patients with abdominal symptoms and headache, it is often difficult to differentiate abdominal migraine from AE because of the overlap of symptoms. The most obvious clinical difference is the duration of the symptoms, hours in migraine (4-72h) compared with several minutes in epilepsy.[6] Thus, EEG as a simple and noninvasive investigation may be helpful in differentiating between the two entities. Patients with AE usually have specific EEG abnormalities, particularly a temporal lobe seizure disorder, although some studies had reported an extratemporal origin (parietal or even frontal).[7,8]

Sustained response to anticonvulsants has been accepted as one of the criteria for the diagnosis of AE. However, there are no recommendations on the choice of the anticonvulsant.

Our patient felt paroxysmal episodes of severe abdominal pain, mostly as an isolated gastrointestinal symptom, without any obvious signs of CNS involvement, such as headaches and loss or alteration of consciousness. With EEG monitoring, these episodes of abdominal pain were identified as a prominent symptom of partial seizure generalized from the right temporal lobe discharges. There was also a positive response to anticonvulsant treatment with carbamazepine and thus our patient fulfilled all the criteria for the diagnosis of AE.

As a conclusion, in patients who experience paroxysms of abdominal pain, nausea and vomiting with or without CNS manifestations, a possibility of AE should be considered after exclusion of more common etiologies for the presenting complaints.
